# Dihydrolipoamide Acetyltransferase AceF Influences the Type III Secretion System and Resistance to Oxidative Stresses through RsmY/Z in *Pseudomonas aeruginosa*

**DOI:** 10.3390/microorganisms10030666

**Published:** 2022-03-21

**Authors:** Haozhou Li, Yushan Xia, Zhenyang Tian, Yongxin Jin, Fang Bai, Zhihui Cheng, Wieslaw Swietnicki, Weihui Wu, Xiaolei Pan

**Affiliations:** 1 State Key Laboratory of Medicinal Chemical Biology, Key Laboratory of Molecular Microbiology and Technology of the Ministry of Education, Department of Microbiology, College of Life Sciences, Nankai University, Tianjin 300071, China; shipinxueyuanlhz@163.com (H.L.); yushanxia1993@163.com (Y.X.); zhenyang_tian@126.com (Z.T.); yxjin@nankai.edu.cn (Y.J.); baifang1122@nankai.edu.cn (F.B.); zhihuicheng@nankai.edu.cn (Z.C.); wuweihui@nankai.edu.cn (W.W.); 2 Department of Immunology of Infectious Diseases, Hirszfeld Institute of Immunology and Experimental Therapy, Polish Academy of Sciences, ul. R. Weigla 12, 53-114 Wroclaw, Poland; wieslaw.swietnicki@hirszfeld.pl

**Keywords:** *Pseudomonas aeruginosa*, AceF, RsmY/Z, sRNA, hydrogen peroxide tolerance

## Abstract

Carbon metabolism plays an important role in bacterial physiology and pathogenesis. The type III secretion system (T3SS) of *Pseudomonas aeruginosa* is a virulence factor that contributes to acute infections. It has been demonstrated that bacterial metabolism affects the T3SS. Meanwhile, expression of T3SS genes is negatively regulated by the small RNAs RsmY and RsmZ. In this study, we studied the relationship between the dihydrolipoamide acetyltransferase gene *aceF* and the T3SS. Our results reveal an upregulation of RsmY and RsmZ in the *aceF* mutant, which represses the expression of the T3SS genes. Meanwhile, the *aceF* mutant is more tolerant to hydrogen peroxide. We demonstrate that the expression levels of the catalase KatB and the alkyl hydroperoxide reductase AhpB are increased in the *aceF* mutant. The simultaneous deletion of *rsmY* and *rsmZ* in the *aceF* mutant restored the expression levels of *katB* and *ahpB*, as well as bacterial susceptibility to hydrogen peroxide. Thus, we identify a novel role of AceF in the virulence and oxidative response of *P. aeruginosa*.

## 1. Introduction

*Pseudomonas aeruginosa* is a Gram-negative bacterial pathogen that causes various chronic and acute infections in humans [1]. The bacterium harbors an arsenal of virulence determinants that play important roles in infections [1]. The type III secretion system (T3SS) is a syringe-like complex that directly translocates effector proteins into the host cell cytosol, which leads to the malfunction or death of the cell [2,3]. The expression of the T3SS genes is activated under Ca^2+^ depletion (e.g., by EGTA) or contact with host cells [2,3]. Four major T3SS effectors have been identified in *P. aeruginosa*, namely, ExoY, ExoT, ExoU and ExoS [2]. ExoU has been shown to function as a phospholipase, which directly damages the host cell membrane and causes rapid necrotic death [4]. *P. aeruginosa* strains with the ability to inject ExoU are usually associated with poor clinical outcomes and increased mortality rates [5].

The T3SS machinery and effector genes are regulated by the master regulator ExsA [6]. The *exsA* gene is located in an *exsC-exsE-exsB-exsA* operon. The transcription of the *exsA* gene is driven by its own promoter (P*_exsA_*) and the *exsC* promoter (P*_exsC_*), which are positively regulated by Vfr and ExsA, respectively [7,8,9]. In addition to transcriptional regulators, small RNAs (sRNAs) are involved in the regulation of T3SS genes [10]. The sRNAs RsmY and RsmZ repress the expression of the T3SS genes by sequestering RsmA, a post-transcriptional regulator that positively regulates the T3SS genes and represses the type VI secretion systems (T6SSs) and biofilm formation [11,12,13]. The expression of *rsmY* and *rsmZ* is controlled by the GacS-GacA two-component system [14], whose function is repressed by RetS and activated by LadS [15]. The sensor kinase GacS phosphorylates the response regulator GacA, which binds to the promoters of *rsmY* and *rsmZ* and activates their transcription [14]. RetS is a hybrid two-component regulator with a sensor kinase domain and two response regulator domains [16]. It has been demonstrated that RetS inhibits the GacS activity through three mechanisms: (1) the siphoning of phosphates from the catalytic domain of GacS; (2) the inhibition of GacS autophosphorylation through direct interaction; and (3) the dephosphorylation of the receiver domain of GacS [16,17,18,19]. A recent study identified host-derived mucin glycans as ligands of RetS. The presence of mucin glycans represses the GacS-GacA two-component system through RetS [20]. Meanwhile, the GacS-GacA two-component system is positively regulated by two hybrid histidine kinases, PA1611 and LadS. PA1611 directly binds to RetS, which counteracts the repressive effect of RetS on GacS [21]. LadS activates the GacS-GacA two-component system by directly phosphorylating GacS [15].

Metabolism plays important roles in bacterial virulence [22]. It has been demonstrated that tryptophan and histidine utilization interfere with the expression of the T3SS genes in *P. aeruginosa* [23,24]. The catabolite repression control protein Crc is required for the expression of the T3SS genes in *P. aeruginosa* [25], indicating a relationship between carbon metabolism and the T3SS. The function of Crc is antagonized by a small RNA (sRNA), CrcZ [26]. In our previous screening for T3SS-related carbon metabolism genes, we found that triosephosphate isomerase (TpiA) affects T3SS gene expression through CrcZ [22]. The isocitrate lyase is required for the expression of the T3SS genes under oxygen-limited conditions [25]. Dacheux et al. found that transposon insertions in genes encoding the pyruvate dehydrogenase (PDH) subunits AceE and AceF (previously called AceA and AceB) resulted in the defective expression of the T3SS genes upon Ca^2+^ depletion, which was restored by expression of *exsA* in trans [27]. A mutation in the E1 subunit (AceA/AceE) of pyruvate dehydrogenase resulted in the defective expression of *exoS* in response to Ca^2+^ depletion in a minimal medium containing 0.1% tryptone and glutamate [28]. In this study, we demonstrate that the dihydrolipoamide acetyltransferase AceF inversely influences the T3SS and bacterial tolerance to oxidative stress through RsmY/Z.

## 2. Materials and Methods

### 2.1. Bacterial Strains and Plasmids

The bacterial strains, primers and plasmids used in this study are shown in Table S1. All strains were cultured in Luria–Bertani (LB) broth with agitation at 200 rpm at 37 °C. Antibiotics were used as follows: for *Escherichia coli*, ampicillin at 100 µg/mL, tetracycline at 10 µg/mL; for *P. aeruginosa*, tetracycline at 50 µg/mL, carbenicillin at 150 µg/mL. Construction of the *aceF* gene deletion mutant in the wild-type *Pseudomonas aeruginosa* reference strain PA14 was performed as described previously [29]. A 1005 bp fragment and a 1152 bp fragment that are upstream and downstream of the *aceF* open reading frame were amplified by PCR, respectively. The PCR products were ligated to the plasmid pEX18Tc. The resultant plasmid was transferred into an *E. coli* conjugation donor strain S17-1 and then transferred to wild-type PA14 by conjugation. The PA14 strains with the plasmid integrated into the chromosome (single-crossover mutant) were selected by tetracycline. The single-crossover mutants were grown in LB overnight and plated on plates with 5% sucrose. The *aceF* deletion mutants (double-crossover mutants) were screened by PCR with primers targeting the coding region of *aceF* (Table S1). Other chromosomal gene mutations were generated in the same way. For the overexpression of *aceF* and *exsA*, the coding regions with their native ribosome binding sequences were amplified by PCR using the PA14 chromosomal DNA as the template with the primers shown in Table S1. The PCR products were cloned into the pUCP20 plasmid in which the transcription of the genes is driven by the *tac* promoter on the plasmid.

### 2.2. Cytotoxicity Assay

Bacterial cytotoxicity was measured by examining the survival of the A549 cells following *P. aeruginosa* infection. A549 cells were cultured in RPMI 1640 medium containing 10% (*v*/*v*) heat-inactivated fetal bovine serum (hiFBS), penicillin (100 mg/mL) and streptomycin (100 mg/mL) at 37 °C with 5% CO_2_. Then, 1 × 10^5^ cells were seeded into each well of a 24-well plate 20 h before bacterial infection. Overnight bacterial cultures were diluted into fresh LB and cultured at 37 °C to an OD_600_ of 1.0. Then, bacterial cells were washed once with PBS and resuspended in PBS. A549 cells were infected with the bacteria at a multiplicity of infection (MOI) of 50. After 3 h of infection, the medium was removed, and each well was washed with PBS twice and stained with 0.25% crystal violet at room temperature for 15 min. An amount of 0.2 mL 95% ethanol was added to each well, followed by incubation for 30 min at room temperature with gentle shaking. Each sample was then subjected to measurement for OD_590_ using a Varioskan Flash multimode microplate reader (Thermo Scientific, Vantaa, Finland).

### 2.3. Murine Acute Pneumonia Model

The animal experiments were in accordance with Chinese National and Nankai University guidelines for animal research. The protocol was approved by the animal care and use committee of Nankai University College of Life Sciences with the permit number NK-04-2012. The animal facility in Nankai University College of Life Sciences has been approved by Tianjin Municipal Science and Technology Bureau with the permission number SYXK 2019-0003. The animal care and use committee of Nankai University College of Life Sciences has been authorized by the local (Tianjin) government to review and approve animal experiment protocols. The animal facility and protocols are inspected by the local government regularly. The murine acute pneumonia model was performed as previously described [30,31]. The bacteria were cultured to OD600 of 1.0. Bacterial cultures were washed twice with PBS and resuspended to 2 × 10^8^ CFU/mL. BALB/c mice (6 weeks, female) were anesthetized with 90 µL of 7.5% chloral hydrate by intraperitoneal injection and then intranasally inoculated with 4 × 10^6^ CFU bacteria. The mice were sacrificed 12 h post infection (hpi) by asphyxiation with carbon dioxide. The euthanasia of mice was performed following the guidelines from the American Veterinary Medical Association (AVMA) for the euthanasia of animals: 2020 Edition (https://www.avma.org/KB/Policies/Documents/euthanasia.pdf, accessed on 10 January 2022). Less than four mice were placed in a cage. Carbon dioxide was introduced with a flow rate of 3–4 L/min until the mice were unconscious. The death of each mouse was confirmed by respiratory and cardiac arrest. The lungs were isolated and homogenized in PBS containing 1% peptone. The homogenate was serially diluted in LB and then plated on LB plates. The plates were incubated at 37 °C for 20 h before colony counting.

### 2.4. Histology

After infection with the indicated strains (4 × 10^6^ CFU/mouse) for 12 h, the lungs of mice were removed and fixed with 4% paraformaldehyde (Servicebio, Wuhan, China) for 12 h, followed by dehydration in ethanol. The lungs were then embedded in paraffin and sectioned, followed by staining with hematoxylin and eosin. The samples were observed with microscopy as previously described [32].

### 2.5. Real-Time qPCR

Bacteria were cultured overnight and then diluted in fresh LB until they reached an OD_600_ of 1.0. Total RNA was isolated using the ZOMANBIO RNA Rapid Extraction Kit (Zomanbio, Beijing, China). cDNA was synthesized with reverse transcriptase and random primers (TaKaRa, Dalian, China). SYBR II green Supermix (TaKaRa, Dalian, China) was used to perform real-time qPCR (RT–qPCR). To ensure the reliability of the results, we used the housekeeping genes *rpsL* and *PA1805* as the internal controls for each RT–qPCR assay [33,34].

### 2.6. β-Galactosidase Assay

Overnight bacterial cultures were diluted in LB and grown to an OD_600_ of 1.0 at 37 °C. A 500 µL volume of the bacterial cultures was resuspended in Z-buffer (8.5 g/L NaH_2_PO_4_, 4.8 g/L Na_2_HPO_4_, 0.75 g/L KCl, 0.12 g/L MgSO_4_, and 0.35% (*v*/*v*) β-mercaptoethanol, pH 7.0). The OD_600_ of the bacterial suspensions were measured. Then, 10 µL of 0.1% SDS and 10 µL of chloroform were added to the bacterial suspension and vortexed for 15 s. Then, the mixture was incubated at 37 °C immediately after adding 100 μL of 4 mg/mL orthonitrophenyl-galactopyranoside (ONPG) (BBI Life Sciences, Shanghai, China). When the color of the mixture turned yellow, the reaction was stopped by addition of a 500 μL volume of 1 M Na_2_CO_3_. OD_420_ of the reaction mixtures was measured. The β-galactosidase activities (Miller units) = (1000 × OD_420_)/T/500/OD_600_; T represents reaction time [35].

### 2.7. H_2_O_2_ Susceptibility Assay

Bacteria were grown at 37 °C to an OD_600_ of 1.0. The bacterial cells were washed twice with PBS. After resuspension in PBS, the bacteria were incubated in the presence or absence of 300 mM H_2_O_2_ for 1 h at 37 °C. The bacterial survival rate = (live bacterial number with H_2_O_2_ treatment)/(live bacterial number without H_2_O_2_ treatment).

## 3. Results

### 3.1. Mutation in the aceF Gene Reduces the Expression of Type III Secretion System Genes

**Figure 1:**
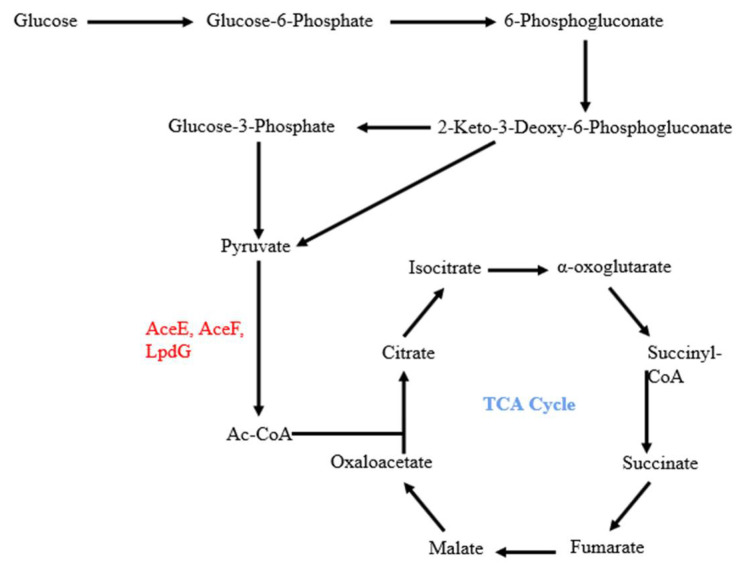
AceE, AceF and LpdG link glycolysis and the tricarboxylic acid (TCA) cycle.

**Figure 2:**
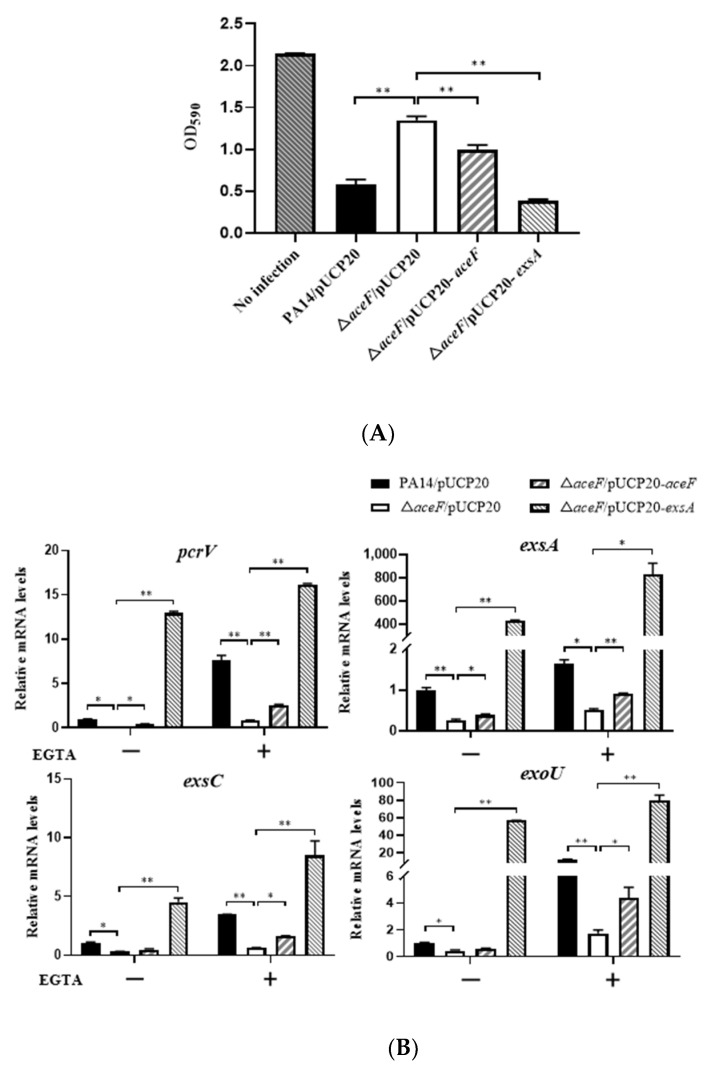
AceF influences the T3SS. (**A**) Cytotoxicity of wild-type PA14, the Δ*aceF* mutant, and the mutant complemented with an *aceF* gene or overexpressing the *exsA* gene. A549 cells were infected with the bacteria for 3 h (MOI = 50). The cytotoxicity was determined by measuring crystal violet-stained cells. (**B**) mRNA levels of the T3SS genes. Bacteria were grown in the presence and absence of 5 mM EGTA for 4 h. The relative mRNA levels of *pcrV*, *exsA*, *exsC* and *exoU* were determined by real-time PCR. Data shown represent mean ± standard error of mean from three samples. * *p* < 0.05; ** *p* < 0.01 by ANOVA.

In our previous screening for metabolic genes that influence bacterial cytotoxicity, we found that an *aceF*::Tn mutant displayed defective cytotoxicity [22]. AceF, AceE and LpdG form the pyruvate dehydrogenase that connects the glycolysis pathway and the tricarboxylic acid cycle (Figure 1). To verify the role of AceF in bacterial cytotoxicity, we constructed an *aceF* in-frame deletion mutant. The deletion of the *aceF* gene reduced the cytotoxicity, which was restored by complementation with an *aceF* gene (Figure 2A). In *P. aeruginosa*, the T3SS plays an important role in bacterial cytotoxicity. In agreement with the reduced cytotoxicity, the mutation of *aceF* reduced the expression of the T3SS genes, including the regulatory genes *exsA* and *exsC*, the structural gene *pcrV* and the effector genes *exoU*, *exoT* and *exoY* (Figure 2B, Figure S1). Complementation with the *aceF* gene or the overexpression of the *exsA* gene restored the expression of these genes (Figure 2B). These results suggest that AceF affects bacterial cytotoxicity by regulating the expression of the T3SS genes.

### 3.2. AceF Affects the Expression of the T3SS Genes through RsmY and RsmZ

**Figure 3:**
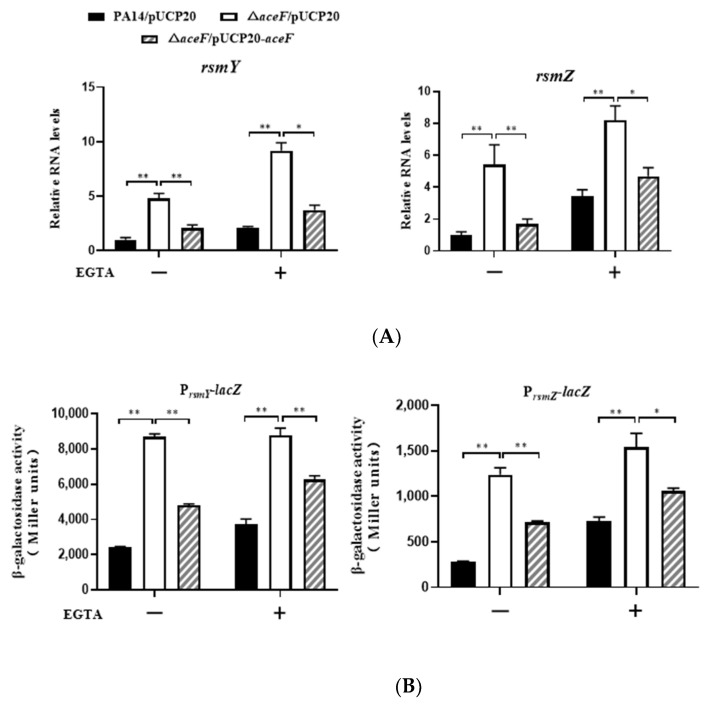
Mutation of the *aceF* gene upregulates *rsmY* and *rsmZ*. (**A**) The bacteria were grown in LB with or without 5 mM EGTA to an OD_600_ of 1. The RNA levels of *rsmY* and *rsmZ* were determined by real-time PCR. (**B**) PA14, the Δ*aceF* mutant and the complemented strain containing the P*_rsmY_*-*lacZ* (**left**) and P*_rsmZ_*-*lacZ* (**right**) transcriptional fusion were cultured in LB with or without 5 mM EGTA to an OD_600_ of 1, followed by determination of the β-galactosidase activity. Data shown represent mean ± standard error of mean from three samples. * *p* < 0.05; ** *p* < 0.01 by ANOVA.

**Figure 4:**
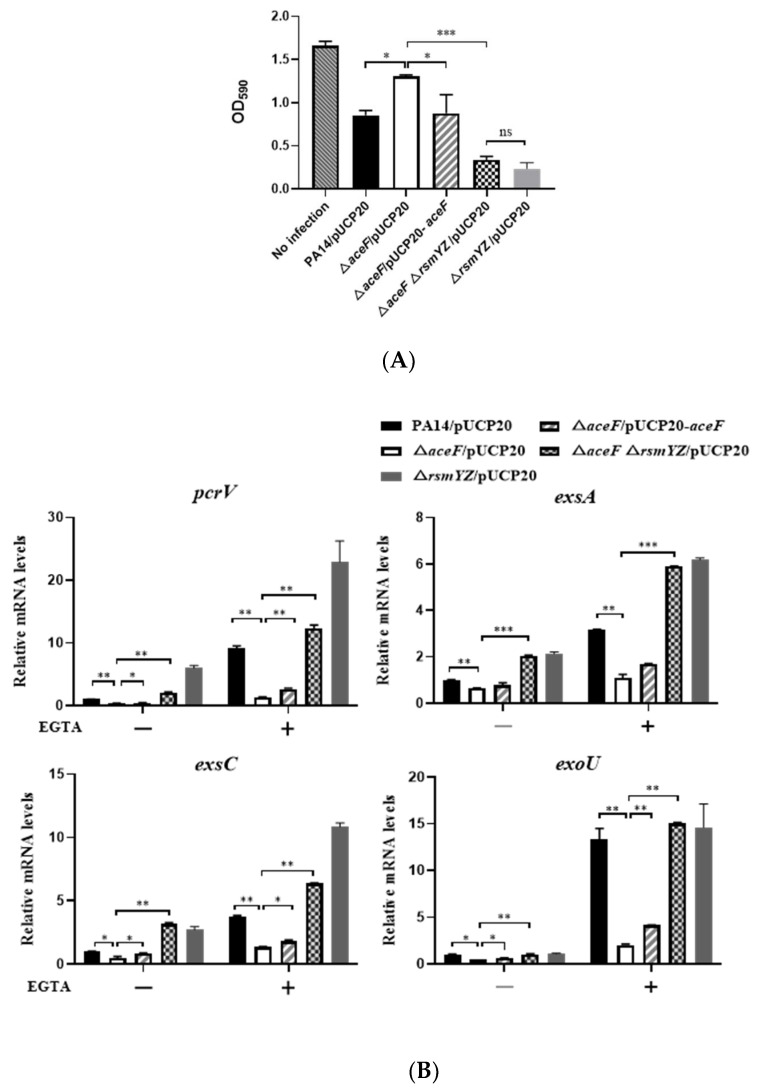
RsmY and RsmZ repress the T3SS in the Δ*aceF* mutant. (**A**) Cytotoxicity of wild-type PA14, the Δ*aceF* mutant, the complemented strain, the Δ*aceF*Δ*rsmYZ* triple mutant and Δ*rsmYZ* double mutant. A549 cells were infected with the bacteria for 3 h (MOI = 50). The cytotoxicity was determined by measuring crystal violet-stained cells. (**B**) mRNA levels of the T3SS genes. Bacteria were grown in the presence and absence of 5 mM EGTA for 4 h. The relative mRNA levels of *pcrV*, *exsA*, *exsC* and *exoU* were determined by real-time PCR. Data shown represent mean ± standard error of mean from three samples. * *p* < 0.05; ** *p* < 0.01; *** *p* < 0.001 by ANOVA.

Given the role of AceF in carbon metabolism, we suspected that CrcZ might be involved in the regulation of the T3SS in ΔaceF. The CrcZ level in the ΔaceF mutant was 1, which is 9-fold lower than that in the wild-type PA14 (Figure S2). Since CrcZ antagonists the function of Crc that positively regulates the T3SS, the downregulation of CrcZ in the ΔaceF mutant is unlikely to cause the defective T3SS. We next examined the expression levels of RsmY and RsmZ. qRT–PCR revealed upregulation of RsmY and RsmZ in the ΔaceF mutant (Figure 3A). By using transcriptional fusions between a lacZ reporter gene and the promoters of rsmY and rsmZ (PrsmY-lacZ and PrsmZ-lacZ), we found that the promoter activities of rsmY and rsmZ were enhanced in the ΔaceF mutant (Figure 3B). To examine whether the upregulation of RsmY/Z leads to the repression of the T3SS, we deleted rsmY and rsmZ in the ΔaceF mutant, which restored the expression of exsA and exoU and bacterial cytotoxicity (Figure 4A,B). However, compared to the ΔaceFΔrsmY/Z triple mutant, the pcrV mRNA levels were higher in the ΔrsmY/Z mutant in the absence and presence of EGTA, and the mRNA level of exsC was higher in the ΔrsmY/Z mutant in the presence of EGTA (Figure 4B). These results indicate that AceF might regulates the transcription or stabilities of pcrV and exsC through an RsmY/Z-independent mechanism. Collectively, these results demonstrate that AceF affects the T3SS and cytotoxicity mainly through RsmY/Z.

### 3.3. Role of AceF in Bacterial Virulence in an Acute Pneumonia Model

**Figure 5:**
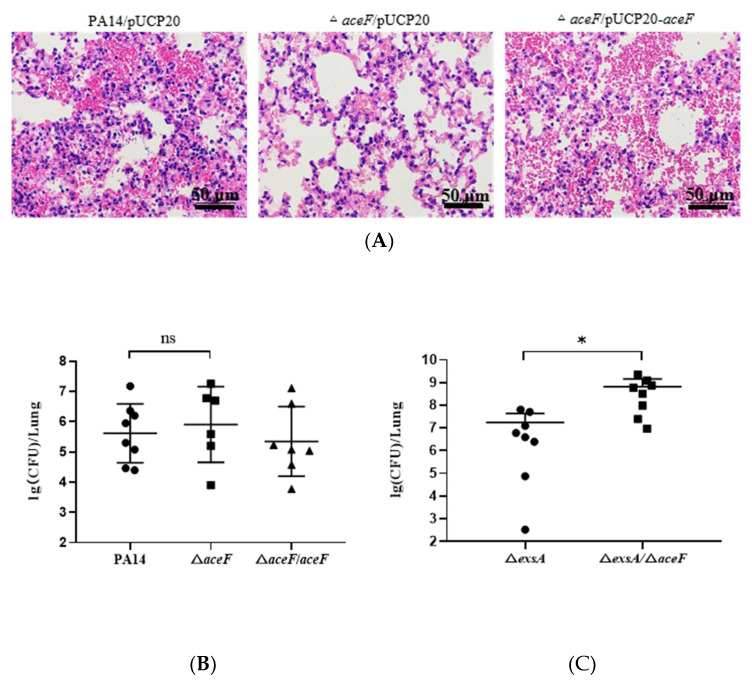
Mutation of the *aceF* gene affects bacterial pathogenesis in a murine acute pneumoniae model. (**A**,**B**) Each mouse was intranasally inoculated with 4 × 10^6^ CFU of bacteria. (**A**) At 12 hpi, the lung sections were observed with a 20× objective lens following hematoxylin and eosin staining. (**B**) Bacterial numbers in the lungs from the infected mice at 12 hpi. (**C**) Each mouse was infected with 4 × 10^8^ CFU of Δ*exsA* or Δ*exsA*Δ*aceF.* At 12 hpi, the bacterial numbers in the lungs were determined. ns, not significant; * *p* < 0.05 by ANOVA.

Since the *P. aeruginosa* T3SS plays an essential role in acute infections, we examined the virulence of the Δ*aceF* mutant in a murine acute pneumonia model by inoculating 4 × 10^6^ CFU of bacteria into each mouse. A pathology analysis of the infected lungs showed that the mutation of *aceF* alleviated the host inflammatory response, which is in agreement with the defective T3SS in the Δ*aceF* mutant (Figure 5A). However, the bacterial loads of the lungs infected by the Δ*aceF* mutant were similar to those infected by wild-type PA14 (Figure 5B). These results indicate that mutations in *aceF* might enhance a mechanism that promotes the bacterial survival independent of the T3SS. To test our hypothesis, we compared the bacterial loads of a Δ*exsA* mutant and a Δ*exsA*Δ*aceF* double mutant. The deletion of *exsA* significantly attenuated the bacterial virulence. At the dose of 4 × 10^6^ CFU per mouse, both the Δ*exsA* and Δ*exsA*Δ*aceF* mutants were cleared at 12 hpi (data not shown). Thus, we increased the dose to 4 × 10^8^ CFU per mouse. The bacterial loads in the Δ*exsA*Δ*aceF* mutant infected mice were higher than those infected by the Δ*exsA* mutant (Figure 5C). Given that the *P. aeruginosa* T3SS preferentially targets bactericidal neutrophils in the acute pneumonia model [36,37,38], these results suggest that the T3SS-defective Δ*aceF* mutant might be more tolerant to the killing mechanisms of neutrophils.

### 3.4. Mutation of the aceF gene Increases Bacterial Tolerance to Hydrogen Peroxide

**Figure 6:**
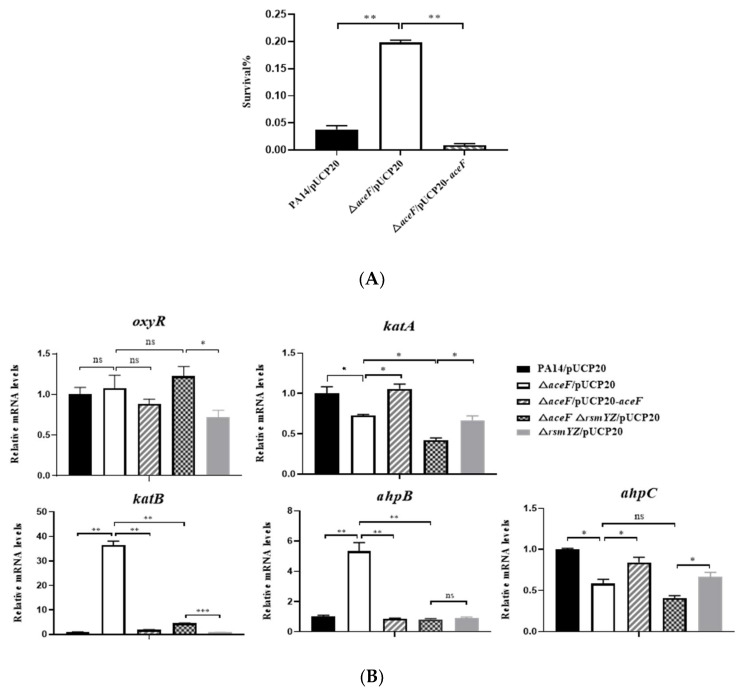

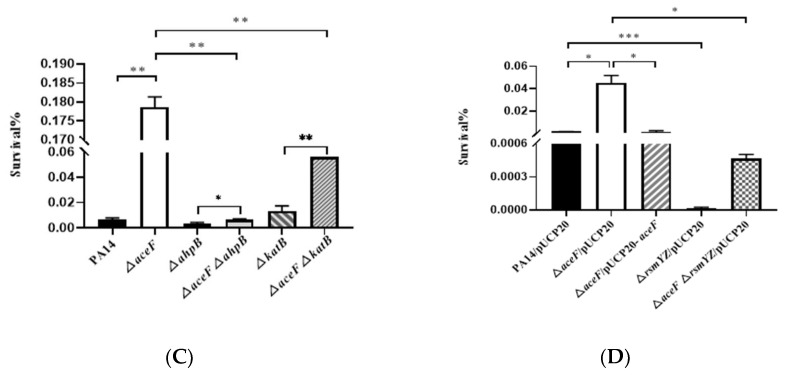
RsmY and RsmZ increase bacterial tolerance to hydrogen peroxide in the Δ*aceF* mutant. (**A**) Survival rates of bacteria following treatment with 300 mM H_2_O_2_ for 1 h. (**B**) The RNA levels of *oxyR, katA, katB, ahpB* and *ahpC* were determined by real-time PCR. (**C**,**D**) survival rates of bacteria following treatment with 300 mM H_2_O_2_ for 1 h. Data shown represent mean ± standard error of mean from three samples. * *p* < 0.05; ** *p* < 0.01; *** *p* < 0.001 by ANOVA.

The generation of reactive oxygen species (ROS) is a major bactericidal mechanism of neutrophils [39,40]. *P. aeruginosa* strains that are defective in the oxidative stress response have been shown to be attenuated in virulence [41,42]. We thus examined bacterial survival following H_2_O_2_ treatment. Compared to wild-type PA14, the Δ*aceF* mutant displayed a higher survival rate (Figure 6A).

To understand the mechanism of the increased tolerance to H_2_O_2_, we examined the expression of genes involved in bacterial oxidative stress response, including the regulator gene *oxyR*, catalase genes *katA and katB* and alkyl hydroperoxide reductase genes *ahpB* and *ahpC*. The mutation of the *aceF* gene resulted in the upregulation of *ahpB* and *katB* and reduced the mRNA levels of *katA* and *ahpC* by 1.4- and 1.7-fold (Figure 6B). The deletion of *ahpB* and *katB* reduced the H_2_O_2_ tolerance of the Δ*aceF* mutant, which reduced the differences in survival between wild-type PA14 and the Δ*aceF* mutant from 26.8-fold to 2- and 4.2-fold, respectively (Figure 6C). These results indicate that the upregulation of *ahpB* and *katB* contributes to the increased H_2_O_2_ tolerance of the Δ*aceF* mutant.

We next examined whether RsmY/Z are involved in the regulation of *ahpB* and *katB* in the Δ*aceF* mutant. The deletion of *rsmY*/*Z* reduced the expression levels of *ahpB* and *katB* (Figure 6B) as well as bacterial survival following H_2_O_2_ treatment (Figure 6D). These results demonstrate that the upregulation of RsmY/Z increases the expression of *ahpB* and *katB* and subsequent H_2_O_2_ tolerance in the Δ*aceF* mutant.

## 4. Discussion

In this study, we found that the mutation of the dihydrolipoamide acetyltransferase gene *aceF* in *P. aeruginosa* resulted in defective T3SS and enhanced H_2_O_2_ tolerance, which is due to the upregulation of RsmY/Z. By using P*rsmY*-*lacZ* and P*rsmZ*-*lacZ* transcriptional fusions, we found that the activities of the *rsmY*/*Z* promoters were elevated in the Δ*aceF* mutant. The transcription of *rsmY/Z* is under the direct regulation of the two-component regulatory system GacS-GacA. However, we did not observe an upregulation of *gacS* or *gacA* in the Δ*aceF* mutant (data not shown). This warrants further studies to examine whether the activity of GacS-GacA is enhanced (e.g., phosphorylation of GacA) or whether other regulatory genes are involved in the regulation of *rsmY/Z*.

*P. aeruginosa* produces catalases and alkyl hydroperoxide reductases to detoxify ROS [43]. The expression of these oxidative response genes is activated by the transcriptional regulator OxyR upon oxidative stresses [44]. OxyR is an LysR-type transcriptional regulator that consists of an N-terminal DNA-binding domain and a C-terminal regulatory domain. The presence of H_2_O_2_ leads to the formation of an intramolecular disulfide bond in the C-terminal regulatory domain, which results in the activation of OxyR [45,46]. In the Δ*aceF* mutant, *ahpB* and *katB* were upregulated, and *ahpC* and *katA* were slightly downregulated. However, the mRNA levels of *oxyR* were similar to wild-type PA14. These results suggest that AceF is unlikely to influence the protein level or function of OxyR. Since the deletion of *rsmY* and *rsmZ* reduced the expression levels of *ahpB* and *katB*, it might be possible that an unknown regulatory gene is involved in the regulation of *rsmY* and *rsmZ* and subsequent *ahpB* and *katB* in the Δ*aceF* mutant. In addition to OxyR, the stress sigma factor RpoS positively regulates the catalase genes *katA* and *katB* [47,48]. In *Pseudomonas fluorescens*, the expression of *rpoS* is activated by GacA and repressed by RsmA [49]. Further studies are warranted to examine whether the mutation of *aceF* affects the level of RpoS. The stringent response has been shown to control the expression of the catalase genes in *P. aeruginosa* and promotes the bacterial survival under oxidative stresses [47,50]. The stringent response is controlled by the molecule guanosine tetra- and penta-phosphate (p)ppGpp [51]. The homeostasis of (p)ppGpp is controlled by two enzymes, namely RelA and SpoT. RelA is a (p)ppGpp synthetase that is activated by amino acid starvation. SpoT functions as both (p)ppGpp synthetase and hydrolase [52]. It has been demonstrated that in *E. coli*, acyl carrier protein (Acyl-ACP) activates the (p)ppGpp hydrolysis activity of SpoT, whereas uncharged ACP activates the (p)ppGpp synthetase activity [53], indicating a connection between carbon metabolism and the stringent response. The pyruvate dehydrogenase connects the glycolysis and the TCA cycle by converting pyruvates to acetyl-CoA that influx into the TCA cycle. Thus, the mutation of *aceF* might impede the TCA cycle, which might affect the activity of SpoT. Further studies are needed to examine whether the stringent response is activated in the Δ*aceF* mutant.

Previously, we found that the mutation of another carbon metabolism gene *eno* results in the downregulation of *ahpB* and *ahpC*, which increases bacterial susceptibility to oxidative stresses [32]. The *eno* encoded enolase is a glycolytic enzyme that reversibly catalyzes the dehydration of 2-phosphoglycerate to phosphoenolpyruvate [54]. In addition, enolase interacts with ribonuclease (RNaseE), polynucleotide phosphorylase (PNPase) and the RNA helicase RhlB to form the RNA degradosome, which plays important roles in RNA processing and degradation [55,56]. Further studies are warranted to elucidate whether the downregulation of *ahpB* and *ahpC* is due to defective assembly of the RNA degradosome or carbon metabolism.

Overall, our results demonstrated that the mutation of *aceF* induces the expression of the sRNA RsmYZ, which represses the expression of T3SS genes and increases bacterial tolerance to H_2_O_2_. Previous and current studies have revealed that interruptions of carbon metabolism in different reactions result in distinct expression patterns of bacterial virulence factors, demonstrating a complex relationship between bacterial metabolism and virulence.

## Supplementary Materials

The following are available online at https://www.mdpi.com/article/10.3390/microorganisms10030666/s1, Figure S1: Expression levels of *exoT* and *exoY*. Figure S2: Expression levels of *crcZ*. Table S1: The bacterial strains, plasmids and primers used in this study [22,29,57,58,59,60].
