# Supplementary material for: Dihydrolipoamide Acetyltransferase AceF Influences the Type III Secretion System and Resistance to Oxidative Stresses through RsmY/Z in Pseudomonas aeruginosa
Source: Microorganisms. 2022 Mar 21;10(3):666. doi: 10.3390/microorganisms10030666 (PMC8950878; doi:10.3390/microorganisms10030666)
Supplement: Supplementary file 1 [file microorganisms-10-00666-s001.zip › microorganisms-1572870-supplementary.pdf]

Table S1. The bacterial strains, plasmids and primers used in this study.

Strain	Description	Source
PA14	Wild-type strain of *Pseudomonas aeruginosa*	[57]
△*aceE*	PA14 deleted of *aceE*	This study
△*aceF*	PA14 deleted of *aceF*	This study
△*rsmY*Z	PA14 deleted of *rsmY* and *rsmZ*	This study
△*ahpB*	PA14 deleted of *ahpB*	This study
△*katB*	PA14 deleted of *katB*	This study
△*aceF*△*rsmY*Z	PA14 deleted of *aceF*, *rsmY* and *rsmZ*	This study
△*aceF*△*ahpB*	PA14 deleted of *aceF*, *ahpB*	This study
△*aceF*△*katB*	PA14 deleted of *aceF*, *katB*	This study
Plasmid		
pEX18Tc	Gene replacement vector; Tc^r	[29]
pUCP20	*Escherichia–Pseudomonas* shuttle vector with a *lac* promoter; Amp^r	[58]
pDN19lacZΩ	Promoterless *lacZ* fusion vector; Sp^r , Sm^r , Tc^r	[59]
pUCP20-*aceF*	Overexpression of *aceF* gene on pUCP20; Amp^r	This study
pUCP20-*exsA*	Overexpression of *exsA* gene on pUCP20; Amp^r	[22]
P*rsmY*-pDN19lacZΩ	*rsmY* gene promoter of PA14 on a promoterless lacZ fusion vector; Sp^r , Sm^r , Tc^r	[60]
P*rsmZ*-pDN19lacZΩ	*rsmZ* gene promoter of PA14 on a promoterless lacZ fusion vector; Sp^r , Sm^r , Tc^r	[60]
pEX18Tc-△*aceE*	Upstream fragment and downstream fragment of the *aceE* gene on pEX18Tc for deletion *aceE*, Tc^r	This study
pEX18Tc-△*aceF*	Upstream fragment and downstream fragment of the *aceF* gene on pEX18Tc for deletion *aceF*, Tc^r	This study
pEX18Tc-△*rsmY*	Upstream fragment and downstream fragment of the *rsmY* gene on pEX18Tc for deletion *rsmY*, Tc^r	This study
pEX18Tc-△*rsmZ*	Upstream fragment and downstream fragment of the *rsmZ* gene on pEX18Tc for deletion *rsmZ*, Tc^r	[59]
pEX18Tc-△*ahpB*	Upstream fragment and downstream fragment of the *aphB* gene on pEX18Tc for deletion *aphB*, Tc^r	This study
pEX18Tc-△*katB*	Upstream fragment and downstream fragment of the *katB* gene on pEX18Tc for deletion *katB*, Tc^r	This study
pEX18Tc-△*exsA*	Upstream fragment and downstream fragment of the *exsA* gene on pEX18Tc for deletion *exsA*, Tc^r	[59]
Primer	Sequence 5'–3'	Purpose
EcoRI-*aceE*-up-F	CGGAATTCCCCCTGGTCCTGGTAGTACTG	*aceE* deletion
BamHI-*aceE*-up-R	CGGGATCCGACGGGATCGAGGTCTTGC	*aceE* deletion
BamHI-*aceE*-down-F	CGGGATCCATCCGACCTACGCCTACGAA	*aceE* deletion
HindIII-*aceE*-down-R	CCCAAGCTTCCTTGATCGAGACGCTTTCC	*aceE* deletion
EcoRI-*aceF*-up-F	CGGAATTCCCGACCTACGCCTACGAA	*aceF* deletion
BamHI-*aceF*-up-R	CGGGATCCGATGCTTTTCACTACCCCG	*aceF* deletion
BamHI-*aceF*-down-F	CGGGATCCTGGCGATCCTCGGTGTGTCC	*aceF* deletion
HindIII-*aceF*-down-R	CCCAAGCTTGGGCCAGGCGGACGATCA	*aceF* deletion

HindIII-*rsmY*-up-F	CCCAAGCTTGTGCCGTTCTGCATCACCAT	*rsmY* deletion
BamHI-*rsmY*-up-R	CGGGATCCGCATCTCTGCGAGGGGGAG	*rsmY* deletion
BamHI-*rsmY*-down-F	CGGGATCCTCGCTGAGCGGTTTCCACA	*rsmY* deletion
SmaI-*rsmY*-down-R	TCCCCCGGGACCAATCCGCCCCAGGTTT	*rsmY* deletion
HindIII-*rsmZ*-up-F	GCCAACAAGCTTCACAACGCCACCGACAAGAG	*rsmZ* deletion
KpnI-*rsmZ*-up-R	ACCCCGGGTACCTTTTTTGCCTGCCGTTTTAC	*rsmZ* deletion
KpnI-*rsmZ*-down-F	GTGTTCGGTACCCGCAGGAGTGATATTAGCGA	*rsmZ* deletion
XbaI-*rsmZ*-down-R	CTCAACTCTAGAGGTTTCTCGCCCCTGTTGAC	*rsmZ* deletion
EcoRI-*ahpB*-up-F	GGAATTCGCTGATAGGCACGCTGACC	*ahpB* deletion
BamHI-*ahpB*-up-R	CGGGATCCCCAGAAGAACAGCACGACGTA	*ahpB* deletion
BamHI-*ahpB*-down-F	CGGGATCCGCCGAGGCACTGTGATCC	*ahpB* deletion
HindIII-*ahpB*-down-R	CCCAAGCTTCAGAGCGAATCGAGATAGCG	*ahpB* deletion
KpnI-*katB*-up-F	GGGGTACCGGGTTCACTCCCTGTATTTCG	*katB* deletion
BamHI-*katB*-up-R	CGGGATCCGAAGACCTTGGCCATGCTC	*katB* deletion
BamHI-*katB*-down-F	CGGGATCCTCTACAAGGCTGACAGCGAC	*katB* deletion
HindIII-*katB*-down-R	CCCAAGCTTGTCGTCAATGAATCAATGGC	*katB* deletion
EcoRI-*aceF*-F	CGGAATTCAGCGGTGGTGGCACGTAA	*aceF* cloning
BamHI-*aceF*-R	CGGGATCCGGTATGACGGGACGCAGTTT	*aceF* cloning
rpsL-F	CAAGCGCATGGTCGACAAGAG	RT-qPCR
rpsL-R	ACCTTACGCAGTGCCGAGTTC	RT-qPCR
exsA-F	CACGTCGGATAATCCTGATT	RT-qPCR
exsA-R	TAGCGGAGAGGCATGAATA	RT-qPCR
exsC-F	ATGGATTTAACGAGCAAGGTCAA	RT-qPCR
exsC-R	GAGGGACAGGGAAGGCAAA	RT-qPCR
pcrV-F	CACGCTCTATGGCTATGC	RT-qPCR
pcrV-R	AAGGTATCCAGATTGCTCAG	RT-qPCR
exoU-F	TCCGGCGGAAATCAATC	RT-qPCR
exoU-R	CTTAGCCATCTCAACGGTAGTC	RT-qPCR
rsmY-F	CAAAGACAATACGGAAACTCAG	RT-qPCR
rsmY-R	GGGGTTTTGCAGACCTCTA	RT-qPCR
rsmZ-F	TACAGGGAACACGCAACC	RT-qPCR
rsmZ-R	TCCTGATGAATCGCCTCC	RT-qPCR
gacA-F	CCTGATGATCGCCAACTG	RT-qPCR
gacA-R	ATAGGTATTCACGGTCTTCG	RT-qPCR
gacS-F	GAGGAAATGCAGCACAAC	RT-qPCR
gacS-R	GTTCTGGATCTCGATGGT	RT-qPCR
oxyR-F	GCTGCTCAACGACAAGA	RT-qPCR
oxyR-R	ATGTGGCGGATGGTCTC	RT-qPCR
katA-F	AAGAGCTATCGGCACATC	RT-qPCR
katA-R	TGGAACTTGACCCAGAAG	RT-qPCR
katB-F	TACAGCCACATGACCAAT	RT-qPCR
katB-R	CTTGAGCACCTGGATGTA	RT-qPCR
ahpB-F	CGTCGTGCTGTTCTTCTG	RT-qPCR
ahpB-R	TTATCCATGCGGTTGTTGT	RT-qPCR

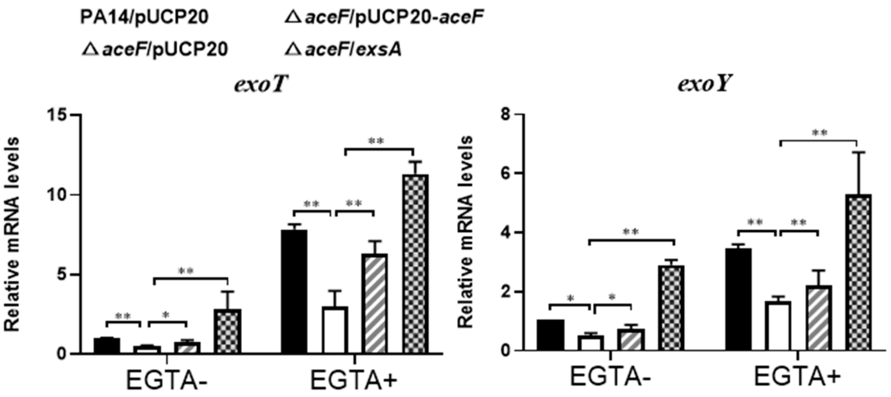

Figure S1. Expression levels of *exoT* and *exoY*. Wild-type PA14, the △*aceF* mutant, the complemented strain and the exsA-over-expression △*aceF* mutant were grown in LB with or without 5 mM EGTA for 4 h. The relative mRNA levels of the T3SS genes were determined by real-time PCR. The 30S ribosomal protein gene *rpsL* and *PA1805* were used as an internal control. Data represent the mean ± standard error of mean of the results from three samples. *, $P < 0.05$; **, $P < 0.01$ by ANOVA.

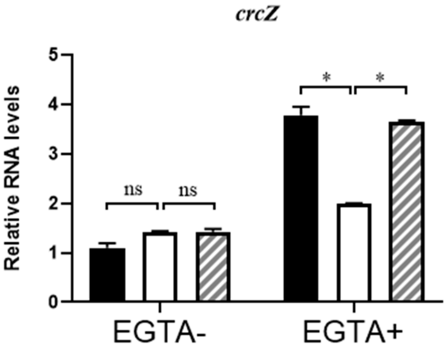

Figure S2. Expression levels of *crcZ*. Wild-type PA14, the Δ*aceF* mutant and the complemented strain were grown in LB with or without 5 mM EGTA to an OD_{600} of 1. The relative levels of *crcZ* were determined by real-time PCR. Data represent the mean ± standard error of mean of the results from three samples. ns, not significant; *, $P < 0.05$ by ANOVA.
